# Circulating Extracellular Vesicles Contain miRNAs and are Released as Early Biomarkers for Cardiac Injury

**DOI:** 10.1007/s12265-016-9705-1

**Published:** 2016-07-06

**Authors:** Janine C. Deddens, Krijn R. Vrijsen, Johanna M. Colijn, Martinus I. Oerlemans, Corina H. G. Metz, Els J. van der Vlist, Esther N. M. Nolte-’t Hoen, Krista den Ouden, Sanne J. Jansen Of Lorkeers, Tycho I. G. van der Spoel, Stefan Koudstaal, Ger J. Arkesteijn, Marca H. M. Wauben, Linda W. van Laake, Pieter A. Doevendans, Steven A. J. Chamuleau, Joost P. G. Sluijter

**Affiliations:** 1 Department of Cardiology, University Medical Center Utrecht, Utrecht, The Netherlands; 2 Department of Biochemistry and Cell Biology, Faculty of Veterinary Medicine, Utrecht University, Utrecht, The Netherlands; 3 UMC Utrecht Regenerative Medicine Center, University Medical Center Utrecht, Utrecht, The Netherlands; 4 Netherlands Heart Institute (ICIN), Utrecht, The Netherlands; 5 Department of Cardiology, Experimental Cardiology Laboratory, University Medical Center Utrecht, Heidelberglaan 100, PO Box 85500, 3508 GA Utrecht, The Netherlands

**Keywords:** Circulating microRNA, Biomarkers, Myocardial infarction, Extracellular vesicles, Exosomes

## Abstract

Plasma-circulating microRNAs have been implicated as novel early biomarkers for myocardial infarction (MI) due to their high specificity for cardiac injury. For swift clinical translation of this potential biomarker, it is important to understand their temporal and spatial characteristics upon MI. Therefore, we studied the temporal release, potential source, and transportation of circulating miRNAs in different models of ischemia reperfusion (I/R) injury. We demonstrated that extracellular vesicles are released from the ischemic myocardium upon I/R injury. Moreover, we provided evidence that cardiac and muscle-specific miRNAs are transported by extracellular vesicles and are rapidly detectable in plasma. Since these vesicles are enriched for the released miRNAs and their detection precedes traditional damage markers, they hold great potential as specific early biomarkers for MI.

**Electronic supplementary material:**

The online version of this article (doi:10.1007/s12265-016-9705-1) contains supplementary material, which is available to authorized users.

## Introduction

Upon myocardial infarction (MI), the heart releases different enzymes, growth factors, and cytokines, which can serve as markers of cardiac injury. Cardiac troponin (cTn) and creatine kinase MB (CK-MB) are the most commonly used biomarkers for MI [1, 2]. Although high-sensitive cTn assays can detect cTn 2–3 h after onset of complaints, guidelines still advise to do serial measurements after 6–9 h for correct diagnosis of MI [1]. Due to the relative late rise of these biomarkers, 10 to 15 % of patients presenting with a MI have a negative blood test upon arrival in the hospital [3, 4]. Delayed confirmation of MI results in increased morbidity and mortality. On the contrary, delayed ruling out of MI prolongs the time spent in the hospital and increases healthcare costs [5]. To guide immediate treatment in the emergency department and to minimize healthcare costs, there is an ongoing need for novel early biomarkers for MI [6].

Increasing evidence suggests that circulating microRNAs (miRNA) can be potential biomarker candidates due to their highly specific elevation in blood upon stress, including MI [3, 7, 8]. MiRNAs are short (∼22 nucleotides) non-coding RNAs [9] that regulate gene expression at a post-transcriptional level [10, 11]. Besides their regulatory intracellular function, they can be released into the extracellular environment where they can contribute to intercellular signaling mechanisms. Circulating miRNAs are closely associated with proteins, lipids, and extracellular vesicles (EV) [12–15].

In a large cohort of patients with suspected acute coronary syndrome (ACS) [3], we found that several miRNAs (miRNA-1, -21, -146a, -208, and -499) are increased in plasma upon injury and have a good diagnostic value to predict MI. However, important detailed information regarding the temporal release profile and potential source and transportation of these miRNAs in the circulation is largely lacking.

EV are the most investigated entities of extracellular miRNA transport and include vesicles derived from the plasma membrane and exosomes, which originate from the endosomal pathway [16]. Exosomes are small lipid bilayer vesicles (30–100 nm) with a density of approximately 1.10–1.17 g/ml [17, 18]. They are enriched with membrane proteins (e.g., CD9 and flotillin-1 [19]) and contain (specific) cellular cargo [17, 20].

Interestingly, high numbers of microparticles and EV are associated with the presence of cardiovascular disease (CVD) [21]. It is demonstrated that the release of EV correlates with the severity of cardiac injury [22]. In addition, plasma EV-packed protein and miRNAs showed potential benefit as biomarkers in the diagnosis of CVD [23, 24]. The combination of both the amount and content of EV in CVD consequently holds great potential for EV as biomarker of MI [21].

Since EV represent major transport vehicles for circulating miRNAs, we assessed the temporal release of extracellular vesicles by the injured myocardium. Moreover, we investigated the potential of EV-linked miRNAs as early biomarkers for MI.

## Methods

Animal experiments were approved by the Animal Ethical Experimentation Committee of Utrecht University and carried out in accordance with the Guide for Care and Use of Laboratory Animals.

### Mouse Model

#### Myocardial Ischemia Reperfusion

**Fig. 1:**
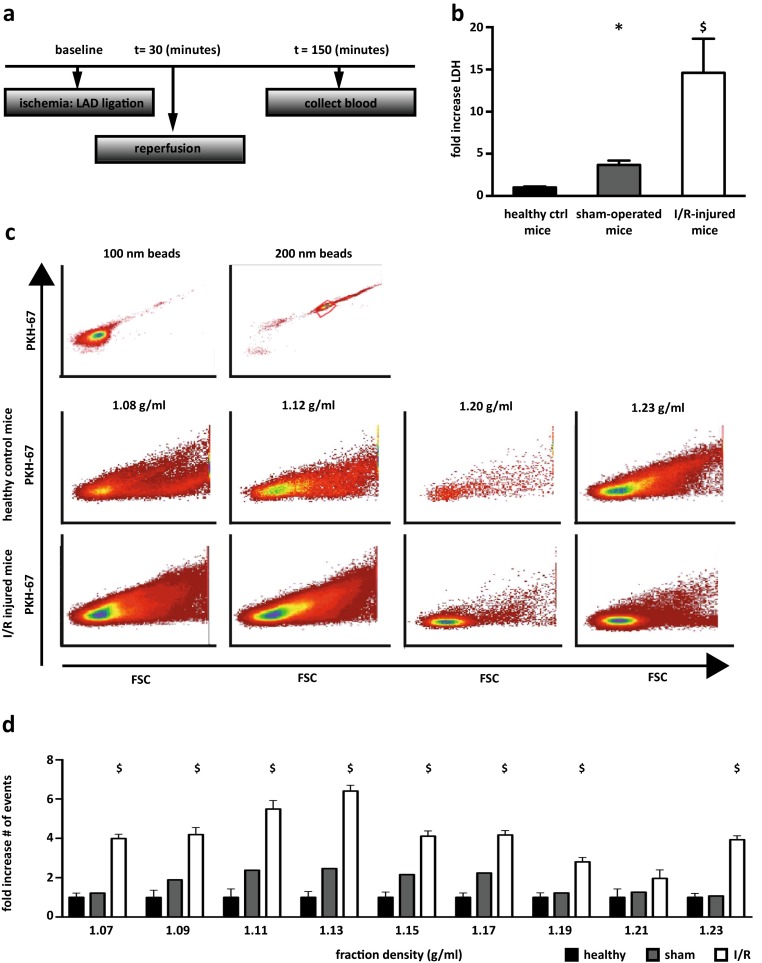
Release of extracellular microvesicles after cardiac ischemia/reperfusion injury in mice. **a** Overview of I/R injury model in C57BL/6 mice. **b** Lactate dehydrogenase (LDH) levels in plasma of healthy control mice (*n* = 5, reference value), sham-operated (*n* = 7), and I/R-injured (*n* = 7) mice at *t* = 150 min. **c** Representative images of high-resolution flow cytometric analysis of isolated PKH67-labeled extracellular microvesicles (EV) from healthy control mice (*middle panels*) and I/R-injured mice (*bottom panels*) in different sucrose gradient fractions. Density scatterplots show reduced wide-angle forward scatter (FSC) plotted against PKH67 fluorescence. **d** Relative time-based quantification of EV from mouse plasma after I/R injury (*n* = 3) and sham operation (*n* = 1) demonstrated a significant increase in vesicle density upon I/R injury, compared to healthy control mice (*n* = 3, reference value) at *t* = 150 min. *Bars* represent mean fold differences, compared to healthy control: *p < 0.05 and $*p* < 0.01

To assess EV release after myocardial infarction in vivo, male C57BL/6 mice (aged 10–12 weeks) were anesthetized intraperitoneally with fentanyl 0.05 mg/kg, midazolam 5 mg/kg, and medetomidine 0.5 mg/kg. Myocardial infarction was induced by ligation of the left anterior descending coronary artery (LAD) as previously described [25]. After 30 min of occlusion, the ligature was removed to allow reperfusion of the myocardium for another 2 h (see Fig. 1a) [26]. For control experiments, plasma was obtained from healthy or sham-operated mice. The sham operations included all of the procedures used for I/R, including length of operation procedure, except the occlusion and successive reperfusion of the LAD. Blood was collected via cardiac puncture of the left ventricle and twice centrifuged at 2000×*g* for 20 min to isolate the plasma fraction.

Operations were performed in three different experiments. The total number of mice included in the study is *n* = 8 (healthy), *n* = 7 (sham), and *n* = 9 (I/R). As the amount of obtained plasma per mice was low and the amount needed for analysis high, pooling of samples was performed for FACS and Western blot analyses.

To assess the extent of cardiac injury, individual plasma levels of total lactate dehydrogenase (LDH) were determined by LDH based toxicology assay kit (Sigma; TOX7), according to manufactures protocol.

#### Langendorff

C57BL/6 mice were injected with heparin 100 IU/kg via the tail vein to prepare for Langendorff retrograde perfusion (*n* = 4) [27]. In short, mice were anesthetized as described above and hearts were quickly excised and placed in ice-cold tyrode buffer solution (NaCl 124 mM; KCl 4.7 mM; MgCl_2_ 1.0 mM; NaHCO_3_ 24 mM; CaCl_2_ 1.3 mM; glucose 11.0; pyruvic acid 5.0 mM, pH = 7.4). Explanted mouse hearts were cannulated through the aortic opening and connected to the Langendorff perfusion system to allow for 2–3 h of retrograde perfusion. The perfusion buffer was kept at 37 °C and was gassed with carbogen (85 % O_2_ + 15 % CO_2_) at a constant pressure of 73 mmHg. For induction of myocardial ischemia, the LAD was ligated. During perfusion, the heart flow-through was collected for isolation of EV.

### Porcine Model of Myocardial Infarction

Female Dutch Landrace pigs (approx. 70 kg, *n* = 6) were anesthetized in the supine position and intubated with an endotracheal tube. The animals were mechanically ventilated by positive pressure ventilation with a mixture of oxygen and air (FiO_2_ 0.5). General anesthesia and analgesia was maintained with midazolam (0.5 mg/kg/h; Roche), sufentanil bromide (2 μg/kg/h; Janssen-Cilag), and pancuronium bromide (0.1 mg/kg/h; Organon) as previously described [28]. During the entire procedure, the electrocardiogram, arterial pressure, and capnogram were continuously monitored. After administration of heparin, MI was created by percutaneous balloon inflation of the LAD below the second bifurcation [29]. Ischemia lasted for 90 min upon which deflation of the angioplasty balloon caused restoration of the blood flow and reperfusion of the tissue. During the procedure, blood samples (citrate) were drawn from the arteria femoralis at several time points: (1) pre-ischemia, (2) 60 min after occlusion, (3) 90 min after occlusion, (4) 1 h after reperfusion, and (5) 2 h after reperfusion (see Fig. 3a).

Citrate blood was centrifuged twice at 2000×*g* for 20 min to isolate plasma, which was stored at −80 °C and used for further analysis. Troponin I levels were analyzed using a clinical chemistry analyzer (AU5811) with a cut-off value of 40 ng/l.

### Extracellular Microvesicles

#### Isolation of Extracellular Microvesicles

To isolate EV, which sediment at ≥10,000×*g*, mouse plasma samples were diluted with PBS (1:1) and centrifuged for 30 min at 2000×*g*, followed by 1 h at 100,000×*g* (Beckmann Coulter LE-80 K Optima, SW60). Pellets with EV were resuspended in PBS and stored for flow cytometric analysis. EV, including exosomes, from mouse and pig plasma and Langendorff perfused hearts were isolated by differential centrifugation, as described before [30, 31]. In short, diluted plasma or flow-through was successively centrifuged at 2000, 10,000, and 100,000×*g*. The resulting 100,000×*g* pellet was resuspended in PBS and centrifuged again at 100,000×*g*. The washed EV pellet was resuspended in a small volume of PBS and the EV protein concentration was determined with the BCA protein assay kit (ThermoScientific). EV were stored at 4 °C until used for electron microscopy and Western blot analysis.

Isolation of pig plasma-derived EV (*n* = 4), to be used for miRNA analysis, was performed by Exoquick isolation, according to the manufacturers protocol (System Biosciences). In short, plasma was centrifuged for 30 min at 10,000×*g* to remove larger vesicles, and 250 μl of the supernatant was added to 63 μl Exoquick. The final pellet was resuspended in diethylpyrocarbonate (DEPC) water and stored in Trizol at −80 °C.

#### Electron Microscopy

Isolated EV were resuspended in phosphate buffer containing 1 % glutaraldehyde (Polyscience; 00216) and subsequently loaded onto formvar/carbon-coated electron microscopy grids. Contrast of the samples was enhanced with uranyl acetate (SPI; 02624-AB). Images were captured using a transmission electron microscope JEOL 1200EX [32, 33].

#### PKH-67 Labeling and Flow Cytometric Analysis

Mouse plasma-derived EV were stained with PKH-67 (Sigma; PKH67GL) to allow for particle detection by flow cytometric analysis [34]. Briefly, EV or an equivalent volume of PBS (control) were dissolved in Diluent C and stained with 7.5 μM PKH-67. After 3 min, PKH-67 labeling was abrogated using 50 μl of vesicle-depleted fetal bovine serum (FBS, centrifuged O/N at 150,000×*g*). To separate PKH-67 labeled EV from unincorporated PKH-67 label, the mixtures were subjected to sucrose density gradient centrifugation, as previously described [30]. Briefly, EV were resuspended in 2.5 M sucrose and layered with decreasing molarities of sucrose before centrifugation for 15 h at 200,000×*g*. After centrifugation, 12 fractions of consecutive densities were collected and diluted ten times in double filtered PBS. As the lower three fractions possibly contain unbound PKH-26, these fractions were excluded from further analysis. EV were analyzed by high-resolution flow cytometry (hFC) using fluorescence threshold triggering as previously described [34, 35]. By means of the low plasma volume of mice, the samples for flow cytometry contained plasma of 2–4 mice in order to obtain measurable numbers of EV.

#### SDS-PAGE and Western Blots

Isolated and sucrose gradient-purified EV were resuspended in 4× laemmli buffer and subjected to SDS-PAGE using pre-casted gels (Novex; NP0335BOX). To compare different experimental conditions (healthy control, sham, I/R injury), samples were corrected for initial plasma volume and loaded in separate lanes. Proteins were transferred to methanol-activated PVDF membranes (Millipore; IPVH00010) to assess the expression of microvesicle-enriched proteins. Membranes were blocked in 5 % milk (Bio-Rad; 170-6404), dissolved in PBS-T20 (0.1 %), and incubated with appropriate antibodies, diluted in 5 % milk-PBS-T20, flotillin-1 (0.4 μg/ml; Santa-Cruz Biotechnology; SC25506), CD9 (0.5 μg/ml, Santa-Cruz Biotechnology; SC53679), and CD63 (1.0 μg/ml, BD; 556019). The proteins were detected with chemiluminescent peroxidase substrate using a Chemi Doc™ XRS+ system (Bio-Rad) and Image Lab™ software.

### Assessment of Circulating miRNAs

#### RNA Isolation and Real Time-PCR (RT-PCR)

RNA from total pig plasma (*n* = 6) and plasma-derived EV (*n* = 4) was extracted using the miRNeasy kit for plasma (Qiagen) and Trizol LS, respectively. Both protocols were performed according to the manufacturer’s descriptions. To correct for isolation variability and to enable comparative analysis of total plasma and plasma EV, C. Elegans miRNA-39 (Quanta Biosciences) was added to the lysis buffer equalized to the starting amount of plasma. RNA quantity and quality were measured with the Nanodrop (NanoDrop Products) and the 2100 Small RNA Assay Bioanalyzer (Agilent). cDNA was synthesized with qScript™ microRNA cDNA Synthesis Kit (Quanta BioSciences), following the manufacturer’s protocol. Quantitative RT-PCR (qRT-PCR) was performed in 12.5 μl duplicate reactions with PerfeCTa SYBR Green SuperMix (BioSciences), the PerfeCTa Universal PCR Primer (Quanta Biosciences), and primers specific for miRNA-1, miRNA-21, miRNA-133b, miRNA-146a, miRNA-208b, and miRNA-499a.

The cycle number that exceeds the fluorescence threshold is the threshold cycle (Ct value). Ct values that exceeded 40 cycles were treated as Ct 42. At missing time points, the average of the other pigs in that specific group was used as the cycle number for that time point. Ct values were normalized by using the average Ct value of the spike-in miRNA.

### Statistical Analysis

Statistical analyses were carried out using GraphPad Prism 6.0 software (GraphPad Software, La Jolla, USA). Differences in miRNA levels were analyzed using a one-way or two-way ANOVA test with a Dunnett’s test for multiple testing corrections. *P* values <0.05 were considered statistically significant. Error bars indicated standard error of the mean (SEM), unless otherwise defined.

## Results

### Ischemia Reperfusion Injury in Mice Leads to the Release of Extracellular Microvesicles into the Circulation

To examine the release of extracellular microvesicles upon extensive cardiac injury, we studied plasma from C57BL/6 mice after 30 min of ischemia followed by 2 h of reperfusion (Fig. 1a). To demonstrate the extent of ischemia/reperfusion (I/R) injury, the level of LDH was measured and showed a 15.5 ± 3.0-fold increase compared to sham-operated and control animals (Fig. 1a). As expected, LDH was also slightly elevated in sham-operated animals (3.9 ± 0.4-fold) compared to healthy controls.

At the same time, EV from plasma of these C57BL/6 mice were isolated and analyzed by hFC [35]. Based on fluorescent labeling with PKH67, individual vesicles were measured by relative time-based quantification. The number of isolated EV from plasma after I/R was increased in all density fractions (Fig. 1c, d) with EV in the fractions with density ranges from 1.11–1.13 g/ml showing the highest increase (5.5 ± 0.4- and 6.4 ± 0.3-fold, respectively; Fig. 1d) in vesicle number (*p* < 0.01). The effect of thoracic surgery is shown by an increase in vesicle number after sham operation, however, to a lesser extent compared to I/R injury (Fig. 1d).

### The Ischemic Myocardium Contributes to the Release of Extracellular Microvesicles after Cardiac Injury

**Fig. 2:**
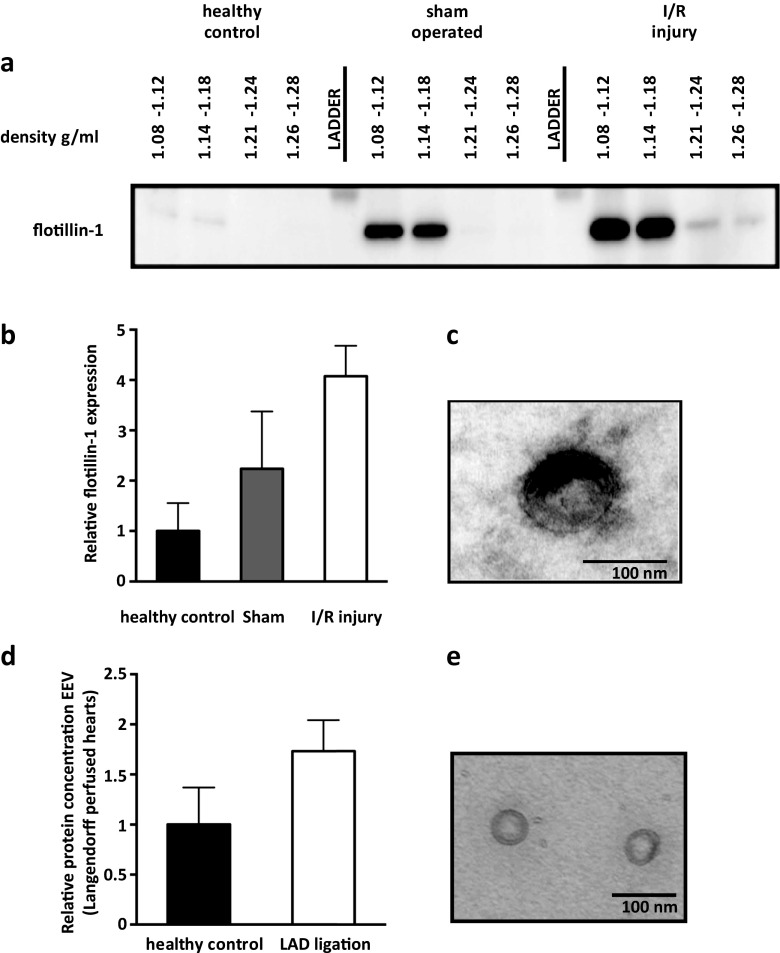
Characterization of mouse plasma-derived extracellular microvesicles. **a** Western blot of flotillin-1 on isolated and sucrose gradient-purified EV in healthy control, sham-operated, and I/R injury mice (*t* = 150 min), *n* = 3, **b** showed that flotillin-1 in EV with a floating density of 1.08–1.12 g/ml was relatively increased. Sample loading was corrected for initial plasma volume and the level of healthy control mice was set to 1. **c** Electron microscopy image of isolated plasma EV. **d** Protein quantification of cardiac-derived (Langendorff perfused heart) EV with and without LAD ligation-induced cardiac ischemia (*n* = 2 vs. *n* = 2). **e** Electron microscopy image of isolated Langendorff perfused heart-derived EV

To shed light on the vesicle distribution after injury, we analyzed sucrose gradient-purified EV by Western blotting and electron microscopy (EM). We observed the presence of the EV-enriched proteins flotillin-1, CD9, and CD63 in these vesicles (Fig. 2a, Online Resource 1a and b for CD9 and CD63, respectively). Flotillin-1 (and CD9) were mainly present in the EV fraction of the sucrose gradient with a density of 1.08–1.18 g/ml. The amount of flotillin-1, as a measure of EV number, was 5.3 (±2.5)-fold increased after I/R compared to healthy controls (Fig. 2b). In addition, isolated vesicles were approximately 100 nm in size and had a lipid bilayer (Fig. 2c).

Next, we aimed at identifying whether the myocardium could be a source of vesicle release upon I/R injury. We harvested EV from healthy and LAD ligation-induced ischemic hearts upon Langendorff perfusion and by that excluded circulating cells as a source of EV. Upon ischemia, by ligation of the LAD ex vivo, the EV protein concentration was increased after 120 min (Fig. 2d), suggestive of increased vesicle release. Lipid bilayer vesicles were observed in the Langendorff flow-through and although these appeared smaller in size than plasma EV (50 nm) (Fig. 2e), they also contained flotillin-1 (Online Resource 1c). These results indicate that the myocardium itself is also able to release EV, including exosomes, which can potentially serve as endogenous carriers for novel biomarkers that are released upon myocardial stress.

Although the release of vesicles could be determined in individual mice for each condition (i.e., control, sham, or I/R injury), their yield was too small to perform miRNA expression analysis. To compensate for the low yield of mouse plasma and to enable temporal analysis, next experiments were performed in a porcine model of myocardial reperfusion injury.

### Plasma-Derived Extracellular Vesicles from a Porcine Model of Ischemia Reperfusion Transport miRNAs Released upon Cardiac Injury

**Fig. 3:**
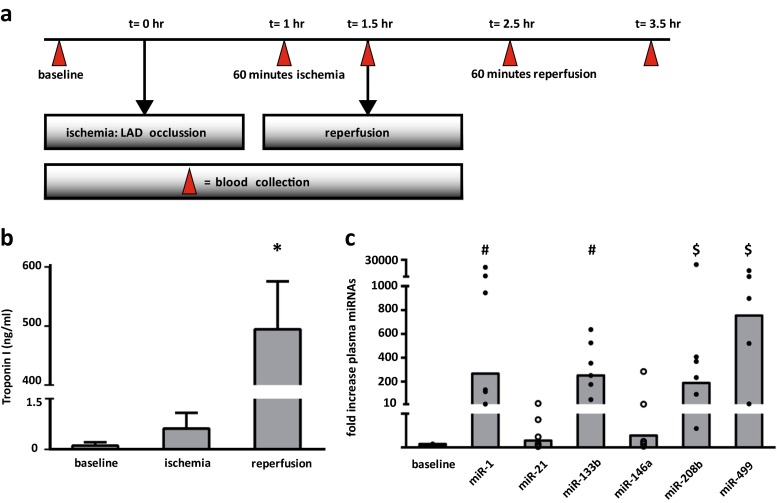
Circulating miRNAs as biomarkers for ischemia/reperfusion injury in a porcine model. **a** Overview of a porcine model of I/R injury by 90 min percutaneous occlusion of the LAD, *n* = 6. **b** Troponin I levels (ng/ml) of plasma at baseline, ischemia (*t* = 1.5 h), and reperfusion (*t* = 2.5 h) demonstrated successful induction of MI. **c** Muscle-specific miRNAs are released in the circulation at *t* = 2.5 h. *Dots* represent fold difference of individual samples, compared to baseline. $*p* < 0.01, #*p* < 0.001

To examine the role of EV as transporters of circulating miRNAs, blood samples were collected at different time points after I/R injury in a porcine model (Fig. 3a, *n* = 6). Successful induction of MI was confirmed by the levels of cTnI in the plasma, which were significantly increased at *t* = 2.5 h, *p* < 0.001 (Fig. 3b). Likewise, levels of well-known circulating miRNAs after I/R injury were analyzed and were significantly upregulated at *t* = 2.5 h (Fig. 3c). The levels of muscle-specific extracellular miRNAs (miRNA-1, -133b, -208b, and -499) in plasma (*n* = 6) were increased up to 750-fold (*p* < 0.0001), thereby demonstrating that miRNA-499 is the most abundantly present miRNA in plasma upon MI. Additionally, the individual levels of miRNA-1, 133b, 208b, and -499 significantly correlated to the levels of cTnI (*R*^2^ = 0.66, 0.61, 0.72, and 0.71, respectively). In contrast to our clinical observations [3], the increase of the inflammatory-related miRNA-21 and miRNA-146 was not statistically significant.

**Fig. 4:**
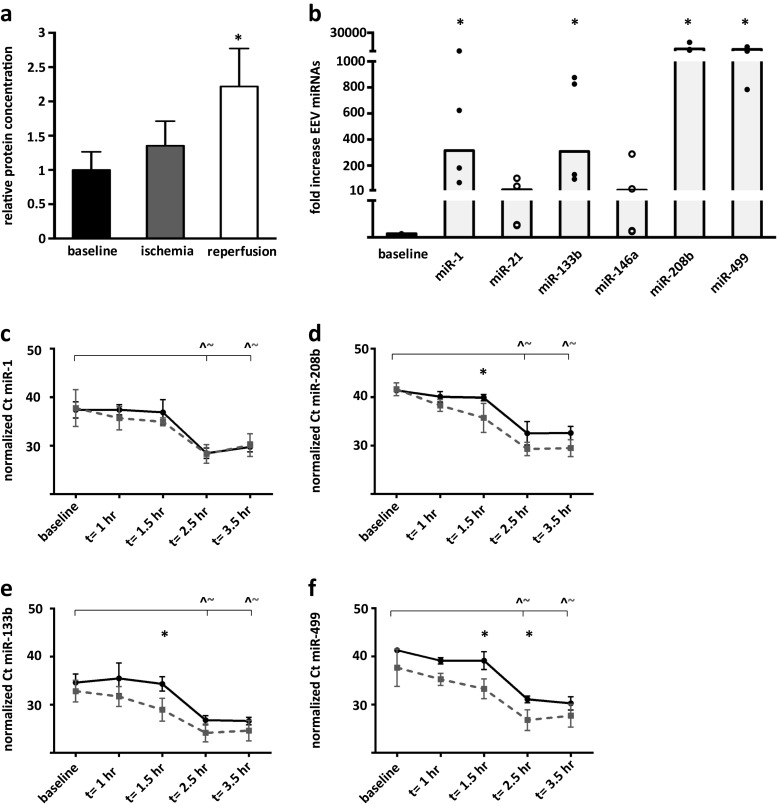
Porcine plasma-derived extracellular microvesicles transport miRNAs after cardiac injury. **a** Release of EV (100,000×*g* pellet) was increased shortly after induction of MI (*t* = 1.5 h). **b** Expression levels of miRNAs in the isolated EV fraction upon I/R injury (*t* = 2.5 h). *Dots* represent fold difference of individual samples, compared to baseline, *n* = 4. **c**–**f** Time dependent analysis of miRNA expression in total plasma (*solid black lines*) and EV fraction (*dotted gray lines*). Data showed an early enrichment of miRNAs in plasma-derived EV at *t* = 1.5 h (miRNA-133b, -208b, and -499) and *t* = 2.5 h (miRNA-133b and miRNA-499). *Error bars* indicate standard deviation, *p < 0.05, and $*p* < 0.01. For **c**–**f**, a significant difference (*p* < 0.05) with baseline is marked with *circumflex accents* for plasma and *tildes* for EV. Additionally, *asterisk* marks a significant difference between plasma and plasma EV at the given time point

As in the previous described murine model, protein levels in the EV fraction increased up to 2.2-fold (±0.6, *p* < 0.05) 60 min after reperfusion (*t* = 2.5 h). Moreover, even after 60 min of ischemia, the amount of EV-derived protein appeared to be increased (1.4 ± 0.4-fold; Fig. 4a).

Since both miRNAs and EV are released in plasma upon cardiac injury, we sought to identify if EV can transport these circulating miRNAs (miRNA-1, -21, -133b, -146, -208b, and -499).

For that reason, the EV fraction was isolated from plasma after I/R injury and the miRNA content was analyzed (Fig. 4b). The plasma vesicles were isolated by Exoquick precipitation after an initial centrifugation step at 10,000×*g* and were termed EV, similar to the vesicles isolated with ultracentrifugation. Parallel to the increase in miRNAs in total plasma after I/R injury, a significant (*p* < 0.01) upregulation of the muscle-specific miRNAs in EV was observed 60 min after reperfusion (*t* = 2.5 h). Levels of miRNA-208b and miRNA-499 were upregulated with the highest amplitude, where miRNA-21 and miRNA-146a showed no significant difference compared to baseline.

To gain insight in the temporal characteristics of miRNA release after I/R injury, a time dependent analysis was performed for both total circulating miRNAs and miRNAs in the EV fraction (Fig. 4c–f, Online Resource 2a, b). Results showed that for both fractions miRNA levels are significantly different at 2.5 and 3.5 h after induction of cardiac injury (*p* < 0.01, compared to baseline). Interestingly, all miRNA curves stabilized after 2.5 h and miRNA-1 levels already decreased at *t* = 3.5 h. This in contrast to the levels of cTnI, which kept increasing between *t* = 2.5 h and *t* = 3.5 h (*p* = 0.03; Online Resource 2c). Finally, results indicated that the miRNA expression levels in plasma-derived EV are higher than total levels in plasma, especially after I/R injury. miRNA expression levels of EV were significant higher than plasma levels at *t* = 1.5 h (miRNA-208b, -133b, -499) and *t* = 2.5 h (miRNA-499) suggestive for (selective) enrichment of miRNAs in plasma EV.

## Discussion

In CVD, an increase in the amount of EV is observed and their content is changed dependent on the severity of disease [21]. Here, we demonstrated that EV are released upon cardiac I/R injury with a substantial contribution of the ischemic myocardium. In addition, serial plasma collection in a porcine model of MI demonstrated a significant increase in both EV and circulating miRNAs upon injury. Cardiac- and muscle-specific miRNAs rapidly increased in plasma 2.5 h after the onset of ischemia while the amount of EV was already increased after 1 h. Interestingly, plasma-derived EV were selectively enriched for miRNA-133b, -208b, and -499, but not for miRNA-1.

Although the release of microparticles by endothelial cells and activated platelets upon MI has been studied before [22, 36, 37], the role of the myocardium in vesicle release is less known. EV release is most commonly analyzed by measuring the protein concentration and expression of exosomal markers of the EV fraction. However, these parameters do not necessarily reflect the number of vesicles [38, 39]. Methods described for individual vesicle quantification are based on nanoparticle tracking analysis [40], tunable resistive pulse sensing [41], or hFC [34, 35]. Using hFC, we quantified EV and provided a detailed analysis of vesicles release upon cardiac injury. Our results confirm the previously described findings of increased EV release upon injury [22] and even augment the data with evidence for myocardial contribution to this process.

To get a clear impression of the characteristics of the EV fraction upon MI, we purified EV by ultracentrifugation and density gradient centrifugation. Based on the observed floating density and membrane markers of plasma EV, it can be suggested that upon cardiac injury, exosome-like vesicles are released [17–19]. Unfortunately, the currently available knowledge and isolation methods are not sufficient to make a clear distinction between the different vesicle sources [42]. However, these findings provide insight in the pathophysiological background and origin of released vesicles, which is crucial for the development of biology-based markers of disease. In addition, profiling the content of EV upon cardiac injury might reveal even more sensitive and specific biomarkers since vesicles can be purified and selective vesicle isolation is possible.

As we described previously [6], EV have an important role in the transport of circulating miRNAs. Several reports show the potential use of miRNAs in the diagnosis and prognosis of cardiac injury [3, 7, 8, 43–47]. Yet, the temporal release profile of miRNAs upon cardiac injury is largely unexplored. Clinical data shows that muscle-specific miRNAs are elevated within 3 h and return back to normal in 3 to 5 days after the onset of MI [6, 48, 49]. Interestingly, in small animal models, the initial increase can already be observed after 1 h [46, 50]. However, sham-operated animals show comparable patterns of which miRNA-499 is only significantly increased. Gidlof et al. [51] described the dynamics of miRNA-1, -133a, -208b, and -499 in a large animal model of I/R injury with a rapid increase in miRNA levels after the occlusion period of 40 min. Although in line with our data, they observed a faster miRNA release and elimination after MI, which can be explained by the difference in occlusion time, extent of cardiac injury, and body weight of the animals.

In contrast with our previous findings in humans [3], no difference in miRNA-21 [52] or miRNA-146a [53] was observed in this study. These miRNAs are differently regulated after MI [52] and are considered to be associated with the inflammatory response [54, 55]. Opposed to patients presenting with a MI, animals used for experiments do not yet suffer from cardiovascular disease prior to the onset of MI and therefore lack any comorbidities. It appears reasonable to speculate that the model we used is not sufficient regarding the levels of the inflammation related miRNA-21 and miRNA-146a and their differential expression is delayed compared to described clinical studies. Parallel to the early rise in cardiac in muscle-enriched miRNAs upon MI, recent evidence suggest that miRNAs can be selectively enriched in EV [24, 56]. Importantly, Jansen et al. [24] demonstrated that miRNA-126 and miRNA-199a were predictive for cardiovascular events only when measured in EV. Additionally, specific transport to the extracellular environment via EV has been shown for miRNA-133 [57]. In accordance with these findings, we have identified that miRNA-133b, -208b, and -499 are enriched in plasma-derived EV. Interestingly, no additional rise in EV-bound miRNA-1 was observed, which is suggestive for selective loading of EV.

The strengths of the present study include that we incorporated several advanced methods to characterize the release of extracellular vesicles. With hFC, we were able to analyze individual vesicles based on membrane staining. Additionally, the experiments with the Langendorff setup enabled us to study the contribution of the ischemic myocardium in vesicle release upon I/R injury. Moreover, we used a large animal model with high clinical relevance to provide novel insights in the temporal and spatial characteristics of circulating miRNAs. By doing so, we could adequately compare the expression levels of miRNAs in total plasma and the EV fraction.

Nevertheless, this study has several limitations. The sham-operated animals were included only once in the hFC analysis (*n* = 1). Although the effect of I/R injury on the release of EV was more pronounced, the thoracic surgery on itself resulted in a low degree of tissue injury, which reinforce the importance of a sham-control group [58]. For this reason, we complemented the data by Western blot-based EV quantification and showed that the majority of EV release is caused by the I/R injury and not the sham procedure. Furthermore, the performed vesicle characterization in the murine I/R model could not be extended with analysis of EV-packed miRNAs. Therefore, we complemented the data in a large animal model, which translates better to the clinic. One should realize the effect heparin has on PCR-based miRNA detection in blood samples and that standardization between samples is therefore essential. However, here we did not observe an effect in time on different miRNAs that were used for normalization [59, 60]. Additionally, only a selected number of miRNAs, based on previously performed studies and their association with MI, were analyzed. Since these selected miRNAs do not fully cover all differentially expressed miRNAs upon MI, conclusions concerning differential packaging of EV are limited. Finally, EV for miRNA analysis were isolated using Exoquick precipitation solution. Although this method is faster than gradient purification, it possibly resulted in co-isolation of RNA of non-EV origin [61]. Identification of miRNAs in EV isolated with different purification methods would therefore be of interest. Furthermore, exploration of miRNAs in other specific plasma fractions, e.g., lipids and proteins, would be of interest to better understand their temporal spatial distribution and their biological context.

In conclusion, we found that the number of EV is increased in different models of I/R injury, faster than cTnI, and are at least partly derived from the ischemic myocardium. We provided evidence that cardiac- and muscle-specific miRNAs are transported by EV and are rapidly detectable in plasma, which suggest that their release is stress-induced. Since EV are selectively enriched for released miRNAs, they hold great potential as specific early biomarkers for MI.

## Electronic Supplementary Material

Below is the link to the electronic supplementary material.

ESM 1 Online Resource 1. Characterization of mouse plasma derived EV. A) CD9 and CD63 (B) are expressed in different sucrose gradient fractions (1.07–1.11 g/ml). C) EV isolated from Langendorff perfusate stain positive for flotillin-1. (EPS 1933 kb)

ESM 2 Online Resource 2. Temporal miRNA analysis. A-B) Expression levels of miRNA-21, miRNA-146a and Troponin I (C) at different timepoints upon I/R injury. Error bars indicate standard deviation, in C a significant difference (p < 0.05) with baseline is marked with ^. * marks a significant difference between t = 2.5 hr and t = 3.5 hr. (EPS 2019 kb)

## Abbreviations

CVD: Cardiovascular disease
CK-MB: Creatine kinase MB
cTn: Cardiac troponin
EV: Extracellular vesicles
hFC: High-resolution flow cytometry
I/R: Ischemia/reperfusion
LAD: Left anterior-descending coronary artery
LDH: Lactate dehydrogenase
MI: Myocardial infarction
miRNA: MicroRNA
